# The Shared Health Appointments and Reciprocal Enhanced Support (SHARES) study: study protocol for a randomized trial

**DOI:** 10.1186/s13063-017-1959-7

**Published:** 2017-05-26

**Authors:** Michele Heisler, Jennifer Burgess, Jeffrey Cass, John F. Chardos, Alexander B. Guirguis, Sean M. Jeffery, Lorrie A. Strohecker, Adam S. Tremblay, Wen-Chih Wu, Donna M. Zulman

**Affiliations:** 1 Veterans Affairs Center for Clinical Management Research, VA Ann Arbor Healthcare System, 2215 Fuller Road, Ann Arbor, MI 48105 USA; 2 Department of Internal Medicine, University of Michigan Medical School, 1600 Plymouth Road, Ann Arbor, MI 48109 USA; 3 Department of Health Behavior and Health Education, School of Public Health, University of Michigan, 1415 Washington Heights, 1700 SPH I, Ann Arbor, MI 48109 USA; 4 VA Northern California Health Care System, 10535 Hospital Way, Mather, CA 95655 USA; 5 Veterans Affairs Center for Innovation to Implementation, VA Palo Alto Health Care System, 795 Willow Road, Menlo Park, CA 94025 USA; 6 VA Connecticut Healthcare System, 950 Campbell Avenue, West Haven, CT 06516 USA; 7 University of Connecticut School of Pharmacy, 69 North Eagleville Road, Storrs, CT 06269 USA; 8 Providence VA Medical Center, 830 Chalkstone Avenue, Providence, RI 02908 USA; 9 Division of General Medicine Disciplines, Stanford University, 1265 Welch Road, Stanford, CA 94305 USA

**Keywords:** SM, Shared medical appointment, Peer support, Disease management, Health plan implementation, Diabetes mellitus

## Abstract

**Background:**

Diabetes shared medical appointments (SMAs) and reciprocal peer support programs have been found in efficacy trials to help adults with diabetes improve their self-management and achieve short-term gains in clinical and patient-centered outcomes. In order to translate this evidence to system-level interventions, there is a need for large-scale, pragmatic trials that examine the effectiveness, implementation, and costs of SMAs and reciprocal peer support across diverse settings.

**Methods:**

The Shared Health Appointments and Reciprocal Enhanced Support (SHARES) study is a multisite, cluster randomized trial that is evaluating the effectiveness and implementation of SMAs with and without an additional reciprocal Peer-to-Peer (P2P) support program, when compared to usual care. The P2P program comprises periodic peer support group sessions and telephone contact between SMA participant pairs to promote more effective diabetes self-management. We will examine outcomes across three different treatment groups: (1) SMAs, (2) SMAs plus P2P, and (3) usual care. We will collect and analyze data over a 2.5-year implementation period at five geographically diverse Veterans Affairs (VA) health systems. The primary outcome is the relative change in hemoglobin A1c over time. Secondary outcomes are changes in systolic blood pressure, antihypertensive medication use, statin use, and insulin initiation over the study period. The unit of analysis is the individual, adjusted by the individual’s SMA group (the cluster). We will use mixed methods to rigorously evaluate processes and costs of implementing these programs in each of the clinic settings.

**Discussion:**

We hypothesize that patients will experience improved outcomes immediately following participation in SMAs and that augmenting SMAs with reciprocal peer support will help to maintain these gains over time. The results of this study will be among the first to examine the effects of diabetes SMAs alone and in conjunction with P2P in a range of real-life clinical settings. In addition, the study will provide important information on contextual factors associated with successful program implementation.

**Trial registration:**

ClinicalTrials.gov, ID: NCT02132676. Registered on 21 August 2013.

**Electronic supplementary material:**

The online version of this article (doi:10.1186/s13063-017-1959-7) contains supplementary material, which is available to authorized users.

## Background

### Rationale

The Centers for Disease Control and Prevention currently estimate that at least one out of three Americans will develop type-2 diabetes in their lifetime [1]. While there are now effective treatments for diabetes, success of these therapies depends on how well patients self-manage over a sustained period of time. Yet, many patients face multiple barriers to effective diabetes self-management (SM) [2, 3]. To meet this challenge, Veterans Affairs (VA) health facilities are working to develop and evaluate low-cost, scalable approaches to increase between face-to-face visit SM support [4].

Shared medical appointments (SMAs) for diabetes bring patients with the same chronic condition together with an interdisciplinary team of providers to provide SM education and support. Several recent meta-analyses of randomized controlled trials (RCTs) of diabetes SMAs have found that SMAs are more effective than usual care in improving hemoglobin A1c levels and systolic blood pressure (SBP) [5–7]. However, most studies of SMAs to date have examined outcomes during, and immediately after, participation in SMAs. There is growing evidence that many patients do not succeed in maintaining SM and clinical improvements achieved through short-term programs [8–11]. Maintaining achieved gains may be especially difficult for patients who lack sustained social support. Both receiving and providing social support is associated with improved SM and clinical outcomes [12–15]. With increasing resource and staff constraints, novel approaches are needed to help patients sustain SM improvements that do not rely exclusively on face-to-face or professionally led programs.

The Peer-to-Peer (P2P) support program, which involves reciprocal peer support in the form of telephone calls between paired participants and periodic patient-directed group sessions, may be a particularly effective complement to SMAs. In an efficacy trial, Heisler et al. found the P2P program to be more effective than nurse care management in improving glycemic control and other diabetes outcomes [16]. The P2P program takes SMAs a step further by providing a long-term infrastructure for the peer support component to continue on a regular basis beyond face-to-face meetings, something especially important for patients who face barriers to accessing care. By augmenting SMAs with the P2P program, SMA participants can continue to work together to improve SM behaviors and sustain gains achieved through the SMAs.

**Fig. 1:**
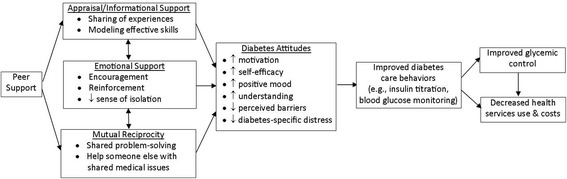
Conceptual framework of hypothesized effects of peer support on diabetes care attitudes, self-management (SM), and clinical outcomes

Figure 1 summarizes the mechanisms through which peer support may enhance chronic disease behaviors and health outcomes.

### Objectives

In order to advance understanding of the value and challenges of implementing SMAs and the P2P program in real-world settings, we designed a multisite pragmatic trial at five VA health care systems in five diverse geographic locations. The study will evaluate whether the addition of the P2P program can improve patients’ SM and outcomes above and beyond the use of SMAs alone—in a combined program called the Shared Health Appointments and Reciprocal Enhanced Support (SHARES) program – over a longer period of time. Our objectives are to:

1. Evaluate the effect of SMAs and SMAs + P2P on diabetic patients’ glycemic control, systolic blood pressure, statin use, and insulin initiation at 6 and 12 months post enrollment compared to usual care.
2. Assess the impact of SMAs and SMAs + P2P on service utilization and patient-centered outcomes, including patients’ satisfaction with VA care, diabetes-specific quality of life and social support at 6 and 12 months post enrollment.
3. Conduct a post-implementation interpretive formative evaluation using constructs from the Consolidated Framework for Implementation Research (CFIR).
4. Estimate program costs using staff effort data obtained throughout implementation.

### Trial design

The study design is a multisite, cluster randomized trial. Randomization will take place in two stages. First, eligible patients will be randomized to usual care versus active treatment. Patients who are invited to participate in SMAs will then be randomized by SMA cohort (the cluster) into one of two conditions: (1) SMAs, or (2) SMAs plus P2P (SHARES). This study involves the collection and analysis of both quantitative and qualitative data during a 23-month implementation period (May 2016 to April 2019). Qualitative data was also collected at VA sites before the study period to identify potential barriers to implementation and will be collected after the study period to evaluate the implementation process, attainment, and sustainability.

Analyses will include all patients randomized to usual care and patients randomized to active treatment. To maintain comparability of the groups, we are including all patients randomly invited to participate in SMAs in the analysis. Thus, the analysis represents a comparison of outcomes of all patients invited for treatment with the outcomes of those eligible patients who were not invited to participate. To restrict our analysis in this comparison to only those patients who agreed to participate in SMAs would result in selection effects among the intervention population and a loss of the advantages of randomization.

Using a mixed-methods approach for data collection and analyses, summative data on clinical outcomes and service utilization will be obtained from VA databases for all patients invited to participate in the programs and those randomly *not* selected for invitation to SMAs. Outcomes will be measured at baseline and at 6 and 12 months post enrollment. When possible, clinical data will be used, especially as data on the clinical measures of interest is already collected as part of patient care. For the two active treatment arms (SMA-only, SMA + P2P), laboratory tests at baseline and 6 months will be ordered through SMA staff as part of the SMA program. As we will have no contact with the usual-care group, we will need to rely on laboratory values ordered as part of their routine clinical care. Thus, we may have more missing values for hemoglobin A1cs, although guidelines recommend checking hemoglobin A1cs at least every 6 months in patients with glycemic control that is not at goal.

For patients enrolled in the SMA-only and SMA + P2P groups who sign a HIPAA authorization, data on patient-centered outcomes will be obtained from surveys. Qualitative data on organizational factors influencing program implementation and characteristics of usual care at each site will be obtained via semistructured interviews of staff at each site. Data on staff time required to implement SMAs and P2P will also be collected to calculate the overall costs of program implementation.

## Methods

### Study setting

Implementation of the programs takes place within the outpatient primary care clinics at five different VA health care systems: Ann Arbor, MI; Palo Alto, CA; Providence, RI; Sacramento, CA; and West Haven, CT. Because this is a study of effectiveness and not efficacy, it is important to implement the programs within the existing context of each of the participating sites; for example, each of the sites already has an established SMA protocol. The protocols differ across sites in SMA frequency and duration of program. However, as a result of our collaboration with the sites during the start-up period, they established comparable programs in terms of group size, key elements included in their SMA curriculum, patient eligibility criteria, patient selection process, and total dose of SMAs (each SMA consists of 6–8 h of meeting time for each cohort). Table 1 refers to the SMA characteristics at each of the sites.

**Table 1:** Shared medical appointment (SMA) characteristics at participating sites

	Ann Arbor	Palo Alto	Providence	Sacramento	West Haven
Session length	2 h	2 h	1 h	1 h	1.5 h
Number of sessions	4	4	8	8	4
Frequency	Every month	Every 2 weeks	Every 2 weeks	Every week	Every 3 months
Total dose	8 h	8 h	8 h	8 h	6 h

### Eligibility criteria

#### Patients

Using automated clinical data, patients are identified as having diabetes if, within the previous 12 months, they had: (1) one hospitalization or two outpatient visits with a diabetes-related *International Classification of Diseases, version 10* (ICD-10) code or (2) at least one prescription for a diabetes medication (excluding glucose-monitoring supplies). To be eligible for the study, diabetic patients need to have had a hemoglobin A1c in the prior 6 months that was greater than 7.5% if aged under 70 years or greater than 8.0% if aged 70 years or older. Using ICD-10 diagnostic codes and automated data, we exclude patients if they had a serious psychiatric illness (bipolar disorder, dementia, schizophrenia, or personality disorders) or active substance abuse (except for nicotine dependence). In addition, patients under the age of 18 years are excluded.

#### VA clinical leadership and staff

As part of the formative evaluations, approximately five to eight VA employees at each of the participating sites have been, or will be, asked to participate in semistructured interviews before, during, and after implementation of SHARES. These employees include the director of primary care, clinicians participating in the SMAs, the P2P group facilitator, and a random sample of two primary care providers. The primary purpose of the interviews is to obtain information on factors likely to influence implementation success.

### Study arms

#### Usual care

Individuals who are randomized to usual care will receive standard primary and specialty care for their diabetes as determined by their clinicians. Patients are randomly assigned to usual care throughout the recruitment period for the purpose of maintaining consistent data collection windows.

#### Active treatment: SMA-only

Individuals in the SMA-only arm are invited to participate in a SMA. At each participating site, the SMA begins with introductions and information sharing, followed by a more open group discussion that also has an educational component. The group discussion facilitates peer interaction and support. In addition to teaching patients about medications and insulin management (e.g., administration, self-monitoring, supplies, self-titration), instruction during the SMAs includes other important areas of diabetes SM such as blood pressure and lipid control, diet, exercise, and stress management. Patients also receive instruction in developing short-term action plans. The patients choose an area of diabetes SM that is important to them to work on and then decide on a concrete step that they feel confident they can take toward meeting this goal over the next week (detailing what they would like to do, how often, how long, where, and ways they will address possible barriers) [17]. The last component of the visit is the clinical component (examination and management) where medication changes and titration are made if necessary.

#### Active treatment: SMA + P2P (SHARES program)

Individuals in the SMA + P2P arm are invited to participate in the SMA, as above. In addition, they are invited to participate in the P2P program between SMA sessions and/or after the completion of the series of SMA sessions. The P2P program consists of patient-directed group sessions and telephone calls between matched SMA participants.

### P2P group sessions

Following an SMA session, participants attend the first P2P group session where they are matched by the P2P facilitator with another SMA participant. Participants are also provided with a brief introduction to peer communication skills to help guide them in providing and receiving peer support. The curriculum used was developed and successfully used in Heisler’s prior RCT, which is based on evidence-based Motivational Interviewing (MI) approaches [18, 19]. Although the emphasis is on enabling patients to assist their partners, MI concepts will also help participants with their own behavioral tasks. The training and participation in peer support is thus expected to promote each patient’s own SM while enhancing their skills in providing support to others; one of the key mechanisms by which peer support may work is to “activate” patients through supporting their peer.

Drop-in group peer support sessions led by a peer facilitator are offered between SMA visits at West Haven (which holds SMAs every 3 months) and at approximately 6-week intervals after patients have finished with the series of SMAs at all other sites. Attendance at the drop-in group sessions is optional. The group sessions provide participants the opportunity for peer support by facilitating discussion of concerns and experiences with living with diabetes, setting and follow-up of SM goals, and shared problem-solving strategies.

### Telephone calls between peers

Participants are encouraged to talk with their partner once per week, with the primary purpose of discussing progress made on SM goals and/or barriers encountered. A research staff member contacts participants 2 weeks after they have been paired to determine if there are any issues with the partners connecting. If a P2P participant reports problems with their partner, both members of the peer support pair are given the option of being assigned to a different partner from other partners requesting reassignment. If participants do not wish to be paired to another partner, they have the option of joining an existing pair who are willing to take on an additional partner or of continuing to participate only in the group sessions. Numbers of partner changes and reasons for changes are tracked.

**Fig. 2:**
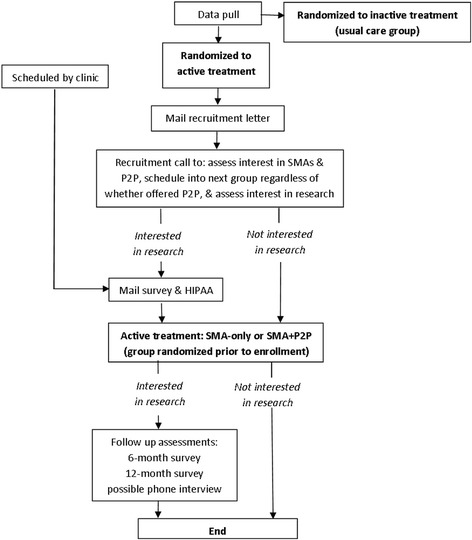
Study flow diagram

Figure 2 outlines the flow of patients through the programs and the research evaluations.

### Outcomes

The principal analysis is a comparison of the effects of participation in the two active treatment arms (SMA-only, SMA + P2P) compared to usual care with respect to the primary outcome of change in hemoglobin A1c level and secondary outcomes of change in SBP, antihypertensive medication use, statin use, and insulin initiation. We will examine the difference between patient average baseline values of hemoglobin A1c and SBP in 6-month windows preceding baseline and following the 6- and 12-month post-enrollment evaluation periods for both active treatment and inactive control groups.

As part of aim 2, we will evaluate change in patient-centered outcomes as reported on patient surveys at baseline and 6 and 12 months post enrollment. These outcomes include service utilization, satisfaction with VA care, diabetes-specific distress, diabetes social support, and diabetes SM behaviors.

### Participant timeline

Table 2 outlines the data collection points of the research evaluation.

**Table 2:** Participant timeline

	Study period			
	Pre-enrollment	Enrollment	6 months post enrollment	12 months post enrollment
Group allocation	X			
Patient surveys		X	X	X
Patient interviews				X
Clinical data (Veterans Administration (VA) database)		X	X	X
Staff interviews	X			X

### Sample size

We chose five VA health systems in which to implement the program based on our estimation of feasibility of implementation, given budget and time constraints. We estimated that we could conduct 10–16 SMA groups per site (equally distributed between SMAs and SMAs + P2P) with 8–14 people per group. At a minimum, this would equate to 560 people in active treatment, including an equal number of inactive controls. In this scenario, we can detect a 0.5% difference in hemoglobin A1c and a 5-mmHg decline in SBP between the active treatment groups and controls.

### Recruitment

Patients can participate in SMAs and P2P even if they elect not to participate in the surveys and interviews. In addition, their medical record data will still be extracted for the overall assessment of changes in clinical measures and utilization in the three arms over the 12-month study period. There will be two methods of recruitment: existing mechanisms (e.g., clinical referral) and data pull.

#### Existing mechanisms

During the start-up period of this project, many of the participating sites established a strong referral base and high participation rate in their SMAs. All patients scheduled for an SMA, who meet our eligibility criteria, are mailed a research packet containing the study information letter, a HIPAA authorization, and the baseline survey. Informed consent is implied by the receipt of a completed baseline survey. If a completed survey has not been received prior to the patient attending an SMA, the patient is approached at the SMA to determine interest in the research study.

#### Data pull

Each quarter, we will pull a list of eligible patients (identified from VA datasets), withholding a set number of random patients reserved for the inactive treatment arm. Invitation letters describing the SMAs, P2P, and the research study are mailed to these patients. Unless patients respond that they do not wish to participate, the letters are followed with a telephone call by research staff to further describe the programs and to schedule interested patients in an upcoming SMA. Those scheduled for SMAs are asked if they are willing to participate in the research study and a research packet is sent to those who express interest in the study.

### Group allocation

Patients are randomized to the usual-care group at the time of each quarterly data pull. All eligible patients in the dataset are assigned a random number and sorted in ascending order. Based on each site’s recruitment target, a set number of patients at the top of this list are then put into the usual-care group. Scheduled SMA groups are randomized to receive P2P by the coordinating center using a random number generator prior to starting participant recruitment. Patients interested in SMAs are scheduled into the next available SMA, regardless of whether or not the participant is interested in P2P. In the case of recruitment through an existing clinical mechanism, the scheduler is not aware of which SMA groups will be offering P2P as an additional optional component. All participants attending an SMA that is being offered P2P will be included in the SMA + P2P analysis group, regardless of whether or not they elect to participate in the optional P2P program.

### Blinding (masking)

Group allocation will be coded in the dataset provided to the data analysts, thus blinding them to treatment allocation.

### Data collection methods

#### VA patients

Data on participating patients is obtained from surveys, interviews, and VA databases. The three surveys (baseline, 6 months, and 12 months) and interviews (for a subset of participants) are obtained for research purposes only. Patients are contacted by mail, with telephone follow-up when needed, to request follow-up survey completion. Existing medical record data for the inactive treatment arm will also be used.

#### VA employees

Data about VA employees is obtained through interviews and used for both program implementation and research purposes.

#### Active treatment fidelity

To the extent that differences will inevitably exist across sites, we are collecting data on differences in execution of the SMA and P2P programs. A member of the research staff attends all SMA sessions for a subset of cohorts to complete a fidelity checklist. The study team discusses any issues in SMA fidelity during biweekly all-team calls. When issues are identified, the research team works with the SMA facilitators to urge concordance with SMA VA national guidelines. Dr. Susan Kirsh, who drafted these guidelines and is a consultant on the project, provides additional training to SMA facilitators when needed. The health behavior coordinators (HBCs) or designee at each of the sites attends the initial P2P group session of a cohort (immediately following an SMA) to complete a fidelity checklist and provide immediate feedback to the facilitators. Additional assessments are conducted of subsequent P2P group sessions at least once every few months. The HBC provides additional training to P2P facilitators as needed to resolve any issues identified during these fidelity checks.

### Data management

Throughout the study, Central Institutional Review Board (CIRB) and HIPAA guidelines are followed to ensure the privacy and integrity of the data collected. To minimize the risk of a breach of confidentiality, we take the following steps. First, each patient recruited for the research component of the study (surveys and possible interview) is assigned a unique study ID. Other than study ID, no identifying information is maintained with patients’ study data, either in hardcopy or electronic formats. All electronic data, including audio files, is stored on a VA server behind the VA firewall. Following transcription of an audio file, all identifying data (e.g., names of other patients, providers, facilities) in the transcript is removed and the audio file is deleted.

Furthermore, study staff members sign a pledge of confidentiality and understand that breach of confidentiality is grounds for dismissal. Study staff members are required to complete annual training on privacy and HIPAA, as well as biannual training on human subject protection. All research findings will be presented in aggregate only.

Survey data is coded and entered by local study team members. Surveys are then sent to the coordinating center, where a second pass data entry is completed and the surveys are securely stored.

### Statistical methods

#### Aim 1

The analysis of hemoglobin A1c values will take the form of a “difference in differences” analysis whereby we will compare final hemoglobin A1c measurements controlling for the baseline hemoglobin A1c measurement among all persons who agreed to participate in one of the two active treatment arms and the inactive control arm. We will use a two-level mixed-effects model with outcome hemoglobin A1c as the dependent variable and including treatment indicator (SMA-only, SMA + P2P, usual care) as the independent variable, baseline hemoglobin A1c and site as covariates, and the group ID as the level-two grouping variable. The coefficient of the treatment variable will indicate the degree by which the change in hemoglobin A1c for the SMA-only and SMA + P2P arms is different from the change in hemoglobin A1c seen in the control arm. An identical analysis will be used for the SBP secondary outcome. Insulin initiation and antihypertensive and statin use will be analyzed using a multilevel ordinal logistic regression with the same basic approach. In a second analysis, we will assess whether there are differences in these outcomes between participants in the SMA-only versus SMA + P2P arms to examine whether there is additional improvements among SMA participants also offered P2P.

All analyses are intention-to-treat analyses. For the first analysis, as we are using an inactive control, in order to get a comparable group we have to define the active intervention population as those people randomly selected to receive an invitation for active treatment. This broadening of the active treatment population will dilute the effect of the intervention as the treatment arm will include the estimated 50% of subjects who decline to participate. In fact, the acceptance rate is itself an interesting parameter to estimate. Furthermore, we will conduct a Complier Average Causal Effect analysis (CACE) in order to estimate the effect of the small group treatment format among the compliers (those who agree to participate). In the second analysis comparing outcomes between the SMA-only and SMA + P2P groups, the comparable groups can be defined as people who agree to participate in whichever type of small group is starting at that time in their site.

#### Approach to missing laboratory data

Although our initial analyses will use only observed data, we will use logistic regression to model patients’ likelihood of having outcome data and define strata within which outcome values are missing at random. We will then stratify patients according to these propensities and randomly sample from the observed outcome distribution and impute these values for missing data within each stratum. Additional sensitivity analyses will also be conducted assuming no change in values for participants for whom we are missing data and using multiple imputation approaches for missing data.

#### Aim 2

To test for differences in resource use by active treatment group status (SMA-only versus SMA + P2P), we will use multivariable models similar to those for aim 1. In addition, since counts of resource utilization are usually quite skewed, alternative modeling techniques, such as Poisson regression, negative binomial regression, or a generalized gamma regression will be used as appropriate. Patients in the SMA-only and SMA + P2P groups who agree to participate in the study will complete the surveys of patient-centered outcomes at baseline, 6 months, and 12 months, allowing for comparisons between outcomes among participants in these two groups. In addition to producing descriptive statistics for all of the scales described above, we will use mixed-effects models (similar to those in aims 1) for continuous outcomes and generalized estimating equations (GEE) for ordinal outcomes with clustering.

#### Aim 3

Analysis of the qualitative data from the post-implementation interpretive evaluation will follow a published methodology for coding and rating CFIR constructs [20]. Once the data has been coded to CFIR constructs, an ordinal value (−2, −1, 0, 1, or 2) will be assigned to each construct at each site. The values represent the perceived magnitude and “role” of each construct in the implementation of the program. The data will be used to construct a matrix for identifying potential correlations between each construct and program outcomes. The findings can be used to develop recommendations for future efforts to disseminate the program, depending on what these factors are, and how they are manifested in the individual sites. We have used this rating process successfully to identify those constructs that appear to be most closely correlated with intervention outcomes in seven different national evaluations of VA programs [21]. The process provides a systematic means of linking constructs to implementation success and allows for the analysis of data across multiple studies using Qualitative Comparative Analysis.

#### Aim 4

To estimate the cost of the SMA and P2P clinical programs, staff are asked to record the time spent on program-related activities during a week-long data collection period. This data is used to estimate the ongoing time involved in running the programs, including time spent on recruiting participants, face-to-face time, and charting. Staff are also asked to estimate the amount of time spent on planning activities required to initially implement SMAs and P2P.

### Data monitoring

As both programs under evaluation, the SMAs and the P2P program, are being offered as part of clinical care at the participating facilities, this study is not required to report to a Data Monitoring Committee.

### Harms

There are no treatments, procedures, or interventions as part of this study. Both patient and employee participants are given a Study Information Sheet with contact information for study staff. Participants are encouraged to contact study staff with any complaints/concerns about the study. We will also ask about any concerns expressed at the biweekly team conference calls. Finally, during the follow-up interviews both patients and staff will be asked to describe any problems that they experienced, so that these can be addressed for future patients. Any complaints or concerns expressed to the study staff by participants, providers, or anyone else affected by this study are immediately reported to the VA CIRB, as will any unexpected events or problems.

### Auditing

Annual audits of regulatory binders and HIPAA authorizations are conducted by the local research compliance officers. Internal audits of the study database are conducted by the coordinating center at quarterly intervals for each site.

## Discussion

### Rationale for undergoing the trial

For diabetic patients, effective diabetes SM is a key determinant of diabetes outcomes. In the face of evidence from efficacy trials that examined diabetes SMAs and reciprocal peer support models separately, there is the need now for large-scale trials and implementation studies that measure real-world impacts of these programs on patient-centered and staff-centered outcomes, costs, and utilization. This study will provide important information on the comparative effectiveness of these programs and barriers that affect the implementation of these programs within existing clinical processes. If augmenting SMAs with a P2P program is found to be effective, this model could be used to improve health outcomes among patients with other chronic conditions requiring significant SM such as depression, chronic pain, and congestive heart failure.

### Dissemination policy

There are three major components to our dissemination plan: (1) incorporation of the program as part of VA’s Patient-aligned Care Teams (PACT) initiative, (2) limiting the resource requirements for the program, and (3) designing a toolkit for implementation (including refinements of the training materials in this proposal’s appendices). Regarding the first component, as participants in the VA Ann Arbor PACT Demo Laboratory, our investigators developed a computer-based “Navigator” system for matching patients to various clinical programs based on their preferences and needs. Patients’ clinical characteristics are obtained from a local registry, and their preferences for types of programs are obtained by the Navigator nurse, all of which are used by a computer-based algorithm to suggest specific programs. The system is designed to emphasize a key aspect of a sustainable care management program—efficiently targeting patients for clinically appropriate programs. Incorporating SMAs and P2P as part of the system will facilitate its inclusion as an important component of PACT and will promote the PACT goal of providing patients with patient-centered care, when they need it and how and where they want it delivered. Disseminating the Navigator at the same time that we promote the dissemination of SMAs and P2P should provide facilities with a way to integrate these programs within the constellation of local programs available for meeting patients’ needs as part of PACT.

In addition, we have designed the P2P program to be an adjunct to SMAs, group diabetes education sessions, or other short-term diabetes or chronic disease SM training programs that are already a part of the patient care process in many VA medical centers. While some investment of effort is required on the part of participating facilities (i.e., training the P2P group facilitators in MI communications techniques, conducting the groups), we believe that this investment is relatively small and should not impede the program’s dissemination in the event that the program is found to be effective.

Finally, we will use our qualitative findings to develop specific recommendations for implementation and to refine materials that can be incorporated into an implementation toolkit.

### Trial status

Patient recruitment started in May 2016 and will continue until April 2018. As of 15 May, 2017, the study has enrolled 304 participants (target = 800) and 238 of those have completed the baseline survey.

## Additional file

Additional file 1: Figure S1. SPIRIT Checklist. (PDF 135 kb)

## Abbreviations

CFIR: Consolidate Framework for Implementation Research
CIRB: Central Institutional Review Board
P2P: Peer-to-Peer
PACT: Patient-Aligned Care Teams
RCT: Randomized controlled trial
SBP: Systolic blood pressure
SHARES: Shared Health Appointments and Reciprocal Enhanced Support
SM: Self-management
SMA: Shared medical appointment
VA: Veterans Affairs
